# Haemodialysis in an emerging centre in a developing country: a two year review and predictors of mortality

**DOI:** 10.1186/1471-2369-12-50

**Published:** 2011-10-02

**Authors:** Udeme E Ekrikpo, Aniema I Udo, Enobong E Ikpeme, Emmanuel E Effa

**Affiliations:** 1 Haemodialysis unit, Department of Medicine, University of Uyo Teaching Hospital, Uyo, Nigeria; 2 Department of Paediatrics, University of Uyo Teaching Hospital, Uyo, Nigeria; 3 Department of Medicine, University of Calabar Teaching Hospital, Calabar, Nigeria

## Abstract

**Background:**

Haemodialysis is the most common form of renal replacement therapy in Nigeria. The high cost of haemodialysis has made optimal therapy of end-stage renal disease difficult in Nigeria. This paper is a review of data collected over two years of provision of dialysis services in a new tertiary hospital in Southern Nigeria.

**Methods:**

This retrospective analysis is done on data obtained from the patient case files and dialysis records in the first two years of provision of dialysis services in our centre. A gender comparison of the patients' baseline sociodemographic, clinical and biochemical was performed and a logistic regression model used to assess the predictors of mortality.

**Results:**

A total of 98 patients had 471 sessions in the two years under review. Males and females had similar characteristics at baseline except for a higher median serum urea in the males. The commonest causes of end-stage renal disease were chronic glomerulonephritis (34.5%), hypertension (32.1%) and diabetes mellitus (17.9%). The main predictor of mortality was under treatment with haemodialysis due to inability to pay for more than a few dialysis sessions.

**Conclusions:**

This study has highlighted the unchanging demographics of our advanced kidney failure patients. Efforts should be aimed at subsidizing the cost of dialysis for our teeming population of dialysis dependent chronic kidney disease patients.

## Background

Haemodialysis is a form of treatment where accumulated solute and fluid are removed from a patient who has total or near-total loss of kidney function using haemodialysis machines which utilize extracorporeal blood lines and artificial kidney called 'dialyzer'[1]. It is indicated for the treatment of Acute Kidney Injury, acute exacerbation of Chronic Renal Failure and End-Stage Renal Disease (ESRD) [1,2]

**Figure 1:**
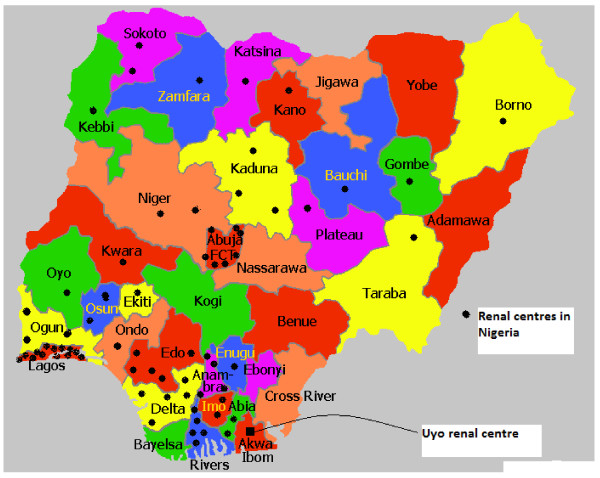
**Map of Nigeria showing the distribution of dialysis centres**.

Haemodialysis is the most commonly used modality of Renal Replacement Therapy (RRT) worldwide.^3 ^A global survey of RRT options showed that 24% of all haemodialysis patients are treated in the United States and 19% in the European Union [3]. In Sub-Saharan Africa including Nigeria, haemodialysis is the most common modality of RRT [4-6]. The other options (peritoneal dialysis and renal transplantation) are largely uncommon due to the extremely exorbitant cost, lack of facilities and manpower, and the predominantly urban location of the renal care centres [4,6,7]. The distribution of dialysis centres in Nigeria with concentration of the centres in the south western part of the country and the Federal Capital territory is as shown in Figure 1.

However, despite the recent increase in the number of haemodialysis centres, the recommendation in the developed world on the optimal dose of haemodialysis is often not achieved for most of the ESRD patients in Nigeria. This is mainly due to the high cost of haemodialysis ($160 per session in Nigeria) and ancillary treatment modalities and interruptions of dialysis services due to frequent breakdown of machines and erratic power supply. For these reasons, it is particularly difficult for patients to maintain regular therapy [4,5].

The National Health Insurance Scheme in Nigeria does not cover the cost of haemodialysis and other renal replacement therapies. As a result, most patients pay out of their pocket. This is an enormous burden for these patients in a country where the Gross Domestic Product (GDP) Per Capita is USD 1,450 and 70% of the population lives below the poverty line [8].

The multiple barriers to accessing RRT underscore the need to identify prevention strategies and methods for fair and equitable access to RRT.

We reviewed our experience over a 2-year period at the Haemodialysis Unit of the University of Uyo Teaching Hospital with emphasis on determining predictors of mortality at baseline.

## Methods

### Setting

This is a retrospective analysis of data at the Dialysis Unit of the University of Uyo Teaching Hospital in southern Nigeria. Set up in January 2008, it is the only dialysis unit in the former south eastern state (comprising Akwa Ibom and Cross River states) with an estimated population of six million and receives referrals from both states and its environs. The unit has three functional dialysis machines. However, there are no machines dedicated to dialyzing patients who have HIV, Hepatitis B and Hepatitis C virus infection. Vascular access for most patients was via femoral catheters. Jugular catheters were not used because this would have added an extra USD 110 to the cost of care. The patients gave informed consent before being commenced on therapy and understood that treatment depended on their ability to pay for this service. Ethical approval for this study was obtained from the University of Uyo Teaching Hospital Human Research Ethics Committee.

### Data extraction/analysis

Data was extracted from patients' medical files and dialysis charts for the period from January 2008 to December 2009. Sociodemographic data, clinical characteristics including blood pressure, clinical diagnosis, age at initiation of dialysis, biochemical and haematocrit levels, dialysis access routes and regularity at dialysis were also extracted. As each patient with ESRD requires at least three dialysis sessions of 12 to 15 hours per week, we defined regularity in our centre as having at least 70% of scheduled dialysis sessions per week (i.e. 2 sessions per week).

Descriptive analysis of the characteristics of the patients was performed using Chi-square for categorical variables and the Wilcoxon rank sum test for continuous variables that were not normally distributed. A logistic regression model was built to determine factors that predicted mortality in our patients. All analysis was done using STATA 10, StataCorp, Texas USA.

## Results

**Figure 2:**
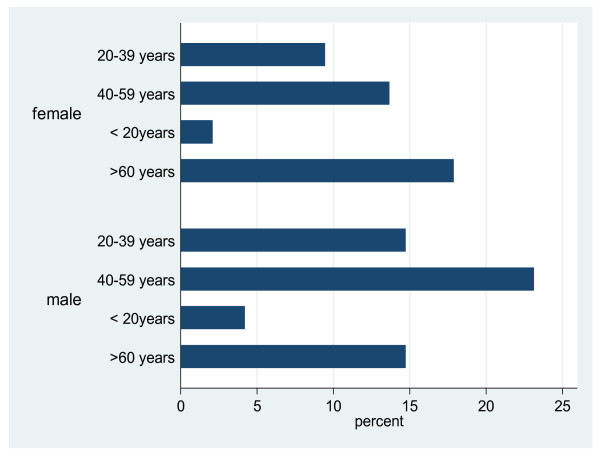
**Age distribution of dialysis patients by gender**.

A total of 98 patients were dialyzed in the period under review. They had a total of 471 dialysis sessions with the median number of sessions per patient being three. Fifty seven percent of the patients were males while 43% were females with a male: female ratio of 1.3:1. The median age at initiation of dialysis was 47.5 years (Males 47 years; Females 48 years) with a range of 12-72 years. Most were in the 20-59 year age group (Figure 2).

Eight four (85.7%) of the patients had chronic kidney disease (CKD) while the remainder had acute kidney injury (AKI). Of those with CKD, 29(34.5%) had chronic glomerulonephritis, 27(32.1%) had hypertensive nephrosclerosis while 15(17.9%) had Diabetic nephropathy. The remainder had adult polycystic kidney disease, obstructive uropathy and sickle cell nephropathy.

The aetiology of acute kidney injury in this cohort included Pre-eclampsia/eclampsia 4(28.6%), drug nephrotoxicity 4(28.6%), sepsis 3 (21.4%), acute glomerulonephritis 2(14.3%), hypovolemic shock from ruptured ectopic pregnancy 1 (7.1%). Two (14.3%) of the AKI patients died: one from disseminated intravascular coagulopathy (DIC) complicating sepsis and the other from progression of uraemia due to lack of funds to pay for more sessions of dialysis.

The clinical and biochemical parameters compared by gender are as summarized in table 1. The mean haematocrit level was 23% (19-29) for males and 22% (20-26) for females. There was a significant difference in the serum urea levels between males and females (27.6 μmol/l versus 22.6 μmol/l, P-value 0.007). Other parameters including serum creatinine and blood pressures were similar. Vascular access for dialysis for 92.6% of patients was via the femoral veins. Only 7 (7.4%) patients had permanent dialysis access (all arteriovenous fistulae). The mean kidney size for individuals presumed to have chronic glomerulonephritis was 8.78 × 3.73 cm for the right kidney and 8.66 × 3.97 cm for the left kidneys.

**Table 1:** Gender Comparison of Clinical Parameters

CLINICAL/BIOCHEMICAL CHARACTERISTIC	MALE	FEMALE	P-VALUE
Serum creatinine (μmol/L)	1318 ± 646	1353 ± 747	0.82
Serum urea (mmol/L), IQR	27.6 (22.2-35.8)	22.6 (18.9-26.8)	0.007
Haematocrit (%)	23 (19-29)	22 (20-26)	0.40
Systolic blood pressure (mmHg)	156 ± 24	161 ± 33	0.46
Diastolic blood pressure (mmHg)	94.5 ± 17.4	98.2 ± 22.2	0.38

Only 11 (13.1%) of our patients had achieved at least 70% of their scheduled dialysis sessions. Seventeen patients received only one session of dialysis, 20 received two sessions; 28 received three sessions; 8 received four sessions; 11 received 5-10 sessions; 4 received 11-15 sessions while only 6 patients received more than 15 sessions in the period of follow up. The highest number of sessions achieved by a patient was 73 sessions in this period. At the end of the second year of providing this service, just about 27% of the patients were still alive. Most of the patients died at home because they could no longer afford haemodialysis.

A multivariate logistic regression model built for the end-stage renal disease patients showed that patients who were able to achieve at least 70% of their scheduled dialysis sessions, had their risk of mortality within the first 2 years of initiating haemodialysis reduced by 93% (Table 2).

**Table 2:** Logistic Regression Model for Predictors of Mortality

VARIABLE	UNIVARIATE ANALYSIS ODDS RATIO (95%CI, P-VALUE)	MULTIVARIATE ANALYSIS ODDS RATIO (95%CI, P-VALUE)
Age (5 years)	1.08 (0.9-1.29, 0.42)	1.09 (0.73-1.61, 0.69)
Serum Creatinine (5 μmol/L)	0.99 (0.99-1.01, 0.67)	0.99 (0.99-1.01, 0.80)
Serum Urea (mmol/L)	1.02 (0.97-1.07, 0.48)	1.05 (0.93-1.19, 0.42)
Packed Cell Volume (%)	1.01 (0.95-1.12, 0.92)	0.85 (0.72-1.01, 0.07)
Systolic blood pressure (mmHg)	1.00 (0.98-1.02, 0.67)	0.99 (0.94-1.03, 0.57)
Diastolic blood pressure (mmHg)	1.00 (0.98-1.03, 0.84)	0.97 (0.92-1.03, 0.28)
**Not regular**	**1**	**1**
**Regular**	**0.24 (0.08-0.67, 0.01)**	**0.07 (0.01-0.56, 0.01)**
Chronic glomerulonephritis	1	1
Diabetic nephropathy	1.86 (0.45-7.73, 0.40)	1.29 (0.04-44.58, 0.89)
Hypertensive nephrosclerosis	1.33 (0.47-3.77, 0.59)	1.40 (0.10-19.52, 0.80)

The model was 92% sensitive and 63.6% specific and the Hosmer-Lemeshow test suggested a good fit for the model. The area under the receiver operator characteristic (ROC) curve for the model was 0.87.

## Discussion

The very high mortality seen in this study is a combination of several factors namely, late presentation, co morbid conditions and inability to pay for the recommended adequate dialysis owing to high cost. Cost of dialysis averages $160 per session in most centres in Nigeria with an estimated GDP of 5.6% for 2009 [9] and lack of health insurance for a majority of people. Indeed, studies in other parts of the country show a similar situation [5,7,10]. The main predictor of mortality in this study was low dialysis frequency due to the inability to pay for more than a small number of dialysis sessions. This has been demonstrated in similar studies in countries with similar health indices [11,12]. An analysis of survival on dialysis in a facility in Ghana shows that duration on dialysis and number of dialysis sessions were very strong predictors of survival among their cohort of patients [13]. Similarly, dialysis frequency and weekly duration of haemodialysis less than 8 hours per week were independent risk factors for mortality in a cohort in Lithuania [14]. Even in resource rich settings, data from the Dialysis Outcomes and Practice Patterns Study (DOPPS) has shown that non adherence among dialysis patients including skipping as little as a dialysis session per month predicts mortality [15]. In our study, even when we considered adequate dialysis as meeting 70% of scheduled sessions which in many cases is still sub optimal, there was a reduction in mortality in those who had at least 70% of their scheduled dialysis sessions. This suggests that more dialysis would have been beneficial. Unfortunately, not many of our patients were able to afford dialysis regularly and for a long time resulting in the high mortality rates seen. Some patients have to commute long distances to access this service. This trend will obviously continue unless there is remarkable expansion of renal care services to many more places, a liberalization of the import regime for dialysis software and consumables to force down the cost of dialysis and the expansion of Health Insurance coverage to include renal replacement therapy especially haemodialysis.

It bears repeating therefore that the high cost of dialysis has militated against access to adequate dialysis. The ethical aspects of initiating dialysis in those with ESRD who presumably may be unable to afford more than a few sessions needs to be further studied in our environment. Often, patients, their relatives or sponsors are provided information on the prognosis and the huge long term cost of RRT before initiating dialysis. In spite of this, they still opt for the initial treatment. Not minding the obvious fact that they may not be able to sustain the therapy in the long term.

The baseline demographic characteristics of our incident patients on dialysis in terms of the age and sex are similar with earlier reports in Nigeria and elsewhere [5,7,10]. It is worrisome that in a developing country such as ours, the economically productive age group is the most affected.

The aetiology of chronic kidney disease still reflects present trends in Nigeria and sub Saharan Africa where chronic glomerulonephritis (CGN) is the commonest cause of CKD [5,10]. Data from Europe and Australia are similar although diabetes rather than hypertension is the second common cause of ESRD. This may reflect lifestyle differences, nutritional variability and a greater control of infectious diseases. The diagnoses of CGN were presumptive based on the relatively young age of the patient, severely impaired serum creatinine levels and markedly shrunken kidney sizes on ultrasound scan.

Predialysis anemia was common in our patients unarguably because of late presentation and poor anaemia management practices before referral for dialysis. Historically, our patients present late preferring to resort to herbal remedies and over the counter medications. Even then, studies from Europe and Asia also show a high prevalence of predialysis anaemia (68% and 75% respectively) in spite of availability of better and affordable renal care services [16-18]. Although anaemia is both a risk factor for CKD as well as a factor that accelerates progression of CKD leading to a worsening of the cardiovascular state, it did not predict mortality in our cohort.

## Limitations

The limitations of this study were firstly, its retrospective design. In establishments such as ours where electronic records are lacking, record keeping can be quite poor. Expectedly, the quantity and quality of data extracted may be suboptimal. Secondly additional baseline information like parathyroid hormone levels, serum calcium and phosphate levels were lacking. These were not routinely done because of the additional cost. This lack precluded our exploration of other risk factors for mortality in our cohort.

## Conclusion

This study has highlighted the unchanging demographics of our advanced kidney failure patients. Frequency of dialysis even as defined in our environment is a strong predictor of mortality. Efforts should be aimed at subsidizing the cost of dialysis for our teeming population of dialysis dependent chronic kidney disease patients who would otherwise not be able to afford regular dialysis.

## Competing interests

The authors declare that they have no competing interests.

## Authors' contributions

UEE and EEI were involved in the initial conception of this manuscript. UEE collected and analyzed the data. AIU, EEI and EEE were involved in the patient care and write up of the manuscript. All authors read and approved the final draft before submission.

## Pre-publication history

The pre-publication history for this paper can be accessed here:

http://www.biomedcentral.com/1471-2369/12/50/prepub
